# Biomolecular mechanisms of epileptic seizures and epilepsy: a review

**DOI:** 10.1186/s42494-023-00137-0

**Published:** 2023-11-15

**Authors:** Komang Trisna Sumadewi, Saktivi Harkitasari, David Christopher Tjandra

**Affiliations:** 1 Anatomy-Histology Department, Faculty of Medicine and Health Science, Warmadewa University, Denpasar, 80235 Indonesia; 2 Neurology Department, Faculty of Medicine and Health Science, Warmadewa University, Denpasar, 80235 Indonesia; 3 Bachelor of Medicine Study Program, Faculty of Medicine, Udayana University, Denpasar, 80234 Indonesia

**Keywords:** Epilectic seizures, Epilepsy, Epileptogenesis, Molecular mechanism

## Abstract

Epilepsy is a recurring neurological disease caused by the abnormal electrical activity in the brain. This disease has caused about 50 new cases in 100,000 populations every year with the clinical manifestations of awareness loss, bruising, and mobility abnormalities. Due to the lack understanding of the pathophysiology behind the illness, a wide variety of medications are available to treat epilepsy. Epileptogenesis is the process by which a normally functioning brain undergoes alterations leading to the development of epilepsy, involving various factors. This is related to the inflammation which is driven by cytokines like IL-1 and tumor necrosis factor-α (TNF-α) leads to neuronal hyperexcitability. Pro-inflammatory cytokines from activated microglia and astrocytes in epileptic tissue initiate an inflammatory cascade, heightening neuronal excitability and triggering epileptiform activity. The blood-brain barrier (BBB) maintains central nervous system integrity through its tight endothelial connections, but inflammation impact BBB structure and function which leads to immune cell infiltration. The mammalian target of rapamycin (mTOR) pathway’s excessive activation influences epileptogenesis, impacting neuronal excitability, and synapse formation, with genetic mutations contributing to epilepsy syndromes and the modulation of autophagy playing a role in seizure onset. The apoptotic pathway contribute to cell death through glutamate receptor-mediated excitotoxicity, involving pro-apoptotic proteins like p53 and mitochondrial dysfunction, leading to the activation of caspases and the disruption of calcium homeostasis. Ionic imbalances within neural networks contribute to the complexity of epileptic seizures, involving alterations in voltage-gated sodium and potassium channels, and the formation of diverse ion channel subtypes. Epileptogenesis triggers molecular changes in hippocampus, including altered neurogenesis and enhanced expression of neurotrophic factors and proteins. Oxidative stress leads to cellular damage, disrupted antioxidant systems, and mitochondrial dysfunction, making it a key player in epileptogenesis and potential neuroprotective interventions. Thalamocortical circuitry disruption is central to absence epilepsy, the normal circuit becomes faulty and results in characteristic brain wave patterns.

## Introduction

Epilepsy is one of the most prevalent neurological conditions that is caused by the abnormal electrical activity in the brain. It is a chronic, paroxysmal, and recurring disease marked by neurobiological, cognitive, psychological, and social factors that collectively contribute to recurrent seizures, alongside enduring risk factors for epileptic episodes [1, 2]. Epilepsy may significantly reduce quality of life, as it limits daily activities, social contacts, and even work chances. Moreover, this disease has caused about 50 new cases in 100,000 populations every year. About 75% of these cases start in childhood indicating the increased vulnerability of brain development to experience seizures [1, 2]. Clinical signs of epilepsy include loss of awareness, mobility abnormalities, sensation (vision, hearing, and taste), mood or cognitive capacities, bruising, and the danger of mortality. A study showed that medication will control the seizures of around 60% to 70% of persons with epilepsy. The general likelihood of epilepsy recurring after experiencing a solitary seizure ranges from 27-71%. Roughly 70% of children will enter a phase where they experience a span of at least 2 years without encountering a seizure. Due to the lack of a complete understanding of the pathophysiology behind the illness, a wide variety of medications are available to treat epilepsy. This circumstance results in a variety of distinct pharmaceutical agents to treat it. Epilepsy characterized by epileptic seizures starts in the gray matter of cortex or subcortex. Epileptic seizures results from an abrupt imbalance between excitation and inhibition in the network of cortical neurons. It starts when neurons exhibit high, coordinated, and sustained neuronal excitability activity.

The term hypersynchrony and hyperexcitability are two different words found in the epilepsy pathophysiology with a related significance among them. The abnormal reactivity of neurons to stimulation input is referred to as hyperexcitability. This condition happens when neurons react too strongly when they receive signals from other cells causing excessive firing of nerve cells in the brain. Neurons commonly start more than two or one electrical activity, which is known as hypersynchrony. This involves several neighboring neurons in aberrant electrical bursts. In this case, a small number of neurons first undergo abnormal stimulation. The normal inhibitory ion exchange and membrane conductance of the synaptic gap are altered, which raises neuronal excitability and synchronization and results in an epileptic seizure. Hyperexcitability is typically brought on by modifications to intrinsic neuronal components and synapse function. According to developments in molecular genetics, several distinct types of human epilepsy are considered to be brought on by mutations in ion channel genes [3–8]. This article aims to comprehensively delve into the intricate molecular underpinnings that give rise to epileptic seizures and the overarching epileptic condition, thereby contributing to a more profound understanding of the etiological complexities inherent to epileptogenesis.

## Epileptogenesis

Epileptogenesis refers to the process through which a normal brain develops alterations that contribute to the formation of epilepsy, alluding brain transformation that was previously functioning normally into a brain that experiences seizures. Thus far, the definition of epileptogenesis remains a topic of ongoing discussion and lacks a universally accepted characterization. This considered involving two processes, including the development of epileptiform processes after brain injury and the process leading to the first spontaneous epileptic seizure. Research shows that neurological alterations during the latent period persist long after epilepsy has been identified and aid in the disease’s development, it means that the mechanisms involved in both starting and developing epilepsy are counted as part of epileptogenesis. The subsequent development or changes that occur following epileptogenesis is called epileptic maturation. The processes of starting epilepsy and what happens afterward are different from each other, it involves the establishment of enduring alterations in neural circuitry and excitability, also often characterized by an increased propensity for recurrent seizures and the manifestation of clinical epileptic symptoms [4]. Prior to the occurrence of the epileptic seizure, the gradual changes of epileptogenesis occurs in neuronal excitability led to changes in interconnections and structure. This include the neurodegeneration, neurogenesis, gliosis, axonal damage or sprouting, dendritic plasticity, breakdown of the BBB, infiltration of inflammatory cells into the brain parenchyma, re-organization of the extracellular matrix, and re-organization of the molecular architecture of individual neurons [9].

Excitability also occurs in the membrane or metabolic components changes of individual neurons. It includes the ictogenesis from specific neurons, neuronal surroundings, and neuronal populations. This results from the suboptimal environmental control, extracellular ion concentration, and neurotransmitters. A neuron can develop into a population of hyperexcitable neurons through anatomical and physiological modifications [5, 10]. By blocking synaptic and voltage-gated inhibitory conductance or activating synapse and voltage-gated excitatory conductance, epileptic activity can be acutely produced. However, it differs from acute seizures in persistent epileptic seizures which are unexpected and less often. In chronic epilepsy, a second mechanism is required to account for the timing of the episodic change from regular activity to an epileptic seizure.

The biological processes, structural changes, and functional alterations play a crucial role in epileptogenesis. The fundamental physiology of an epileptic seizure includes the instability of the cell membrane or the surrounding or neighboring supporting cells. A contributing factor to cell membrane instability involves the deficiency of adenosine triphosphate (ATP) acetate associated with ion transport or anomalies in potassium ion conductance. Some examples of neurotransmitters that can boost excitability and expand neuronal activity are glutamate, aspartate, acetylcholine, norepinephrine, histamine, corticotropin-releasing factor, purines, peptides, cytokines, and steroid hormones. At the same time, dopamine and γ-amino butyric acid (GABA) restrict neuronal activity and growth. During an epileptic seizure, there is an increased blood flow requirement to the brain, facilitating the transportation of carbon dioxide and metabolic substrates essential for neuronal activity. Various forms of epilepsy are associated with mutations in a multitude of genes. It encompasses several types that are correlated with genes encoding protein subunits of voltage-sensitive and ligand-activated ion channels [4, 8].

Epileptic seizures and epilepsy arise from a multitude of causes and sustained through a process of positive reinforcement, where an initial imbalance between neural inhibition and excitation triggers further imbalances. The initial triggering activity not only initiates the seizure but also fosters ongoing neural excitation while simultaneously inhibiting the natural restraining mechanisms. This self-perpetuating cycle is facilitated by positive feedback, effectively prolonging the epileptic seizure once it has been initiated [11]. Epileptogenesis is influenced by factors including oxidative stress, neurochemical alterations in the brain due to neurotransmitters and ion channels, fluctuations in ion concentration, variations in cell surface receptors, and the presence of inflammation. Some targets in epileptogenesis include the mammalian target of rapamycin (mTOR), P-glycoprotein (P-gp), and mutations in voltage-gated ion channels like hyperpolarization-activated cyclic nucleotide-gated (HCN) channels and Kv7 channels (or M-channels), Na-K-2Cl cotransporter isoform 1 (NKCC1) [8, 12].

## The role of inflammation in epileptic seizure and epilepsy

**Fig. 1:**
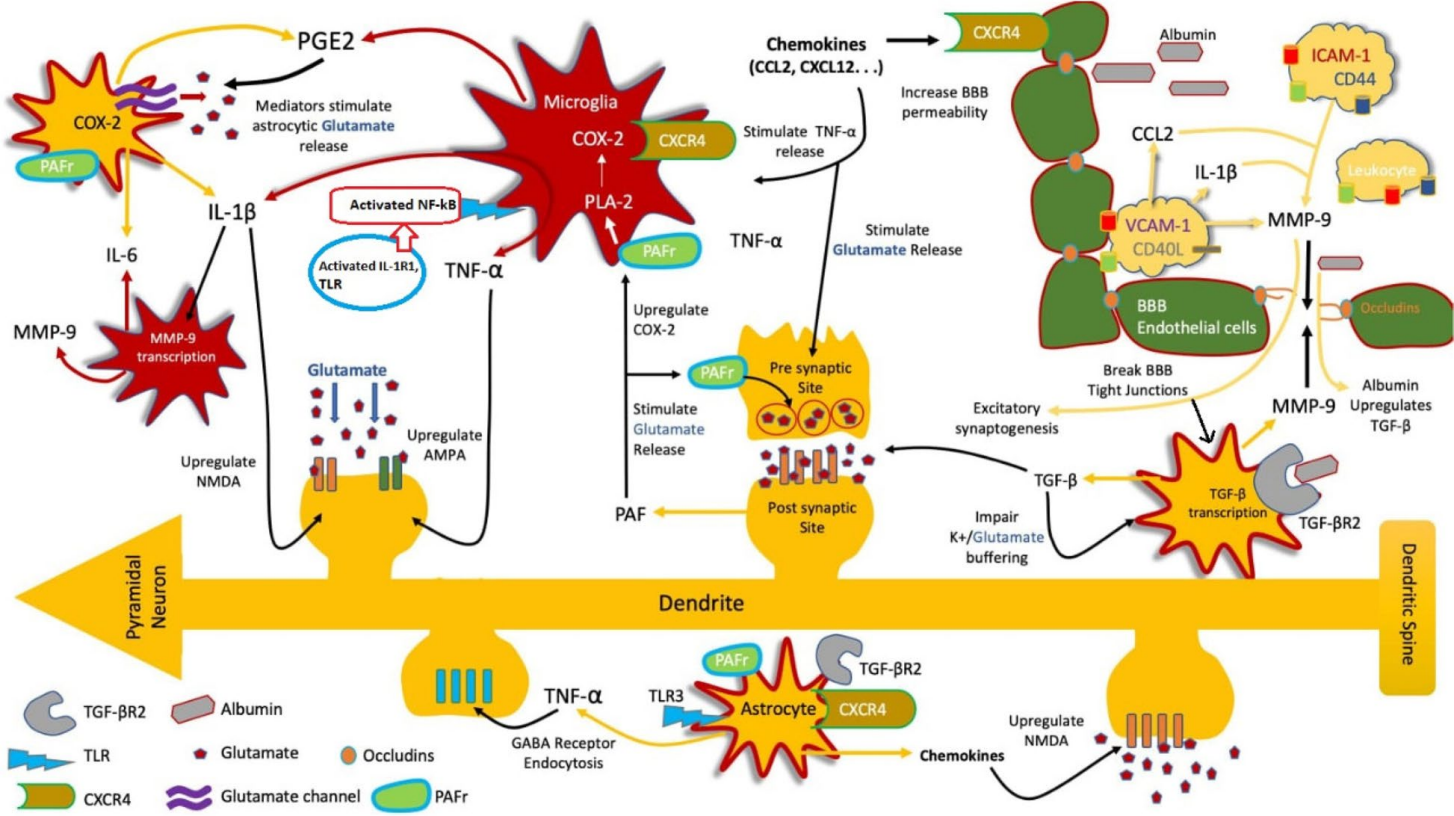
The role of inflammation in epileptic seizure and epilepsy. A cascade of events unfolds after brain injury that triggers central inflammation and alters normal neuronal connections in the hippocampus. Meanwhile, systemic inflammatory diseases cause inflammation in the peripheral tissues, accelerating the accumulation of inflammatory agents. This dual inflammation, both in the peripheral and central systems, contributes to the weakening of the protective BBB by enhancing the levels of inflammatory agents. This compromised barrier permits the infiltration of immune cells, initiating heightened neuronal activity and further amplifying the production of inflammatory agents. The uncontrolled inflammation in both peripheral and central regions, coupled with the compromised blood-brain barrier, sets the stage for structural changes in synaptic connections within the hippocampus. These processes culminate in the progression of epilepsy. Adapted from Rana and Musto [13]

Inflammation indeed plays a significant role in the progression of epileptogenesis. It reduces the threshold for the initiation of epileptic seizures, and it becomes further exacerbated under conditions of SE [8]. Death of neuronal cells, malfunction of synapses, and the onset of hyperexcitability are all symptoms of brain injury brought on by concussions, hypoxia, or febrile convulsions. The release of cytokines, chemokines, lipid mediators, and protectins into the neuronal environment indicates that brain trauma has caused an organized cascade of biological processes. Depending on the part of the brain that is affected, dysregulation of these mediators and the expression of their receptors might result in clinical symptoms that include permanent neuronal injury [13] (Fig. 1).

### Brain inflammation in people with epilepsy

Epileptogenesis is closely linked to high degree of inflammation in the neural tissue’s milieu both acutely and persistently. The central nervous system may be the source of the inflammatory response with possible damage to the BBB. This inflammation can spread from the central nervous system into the systemic circulation, and exacerbations of pre-existing chronic neuroinflammation may also occur as a result of acute neuroinflammation [13]. Small amounts of the cytokine interleukin-1 (IL-1) are produced by activated astrocytes and microglia and expressed in the human central nervous system, these levels are increased during pathogenic processes. These cytokines cause astrocytes to consume less and release more glutamate, resulting in neuronal hyperexcitability. IL-1 is thought to cause epileptic seizures by increasing N-methyl-D-aspartate (NMDA) receptor expression in post-synaptic cells through activation of the GluN2B subunit of the NMDA receptor [13–15].

Active astrocytes and microglia emit the pro-inflammatory cytokine tumor necrosis factor-α (TNF-α). Several research shows that TNF-α controls N-cadherin which is essential for the development and structure of excitatory and inhibitory synapses. TNF-α also boosts glutaminase production at gap junctions, which in turn causes microglia to release more glutamate. TNF-α also increase the the expression of α-amino-3-hydroxy-5-methyl-4-isoxazolepropionic acid (AMPA) receptors, which amplifies glutamatergic transmission and results in excessive calcium ion influx and neurotoxicity. It also triggers GABA receptor endocytosis which reduces the inhibitory excitability, and results in significant alterations in excitability [13–15]. Another multifunctional cytokine that will increase along with the increment of TNF-α, IL-1β, and interferon-γ (IFN-γ) is the interleukin-6 (IL-6) which controls inflammation as immunological responses. The production of IL-6 in the central nervous system remains in low level. However, it can be boosted by the activation of astrocytes and microglia. The increasing levels of IL-6 expression reduces long-term potentiation (LTP), inhibits hippocampus neurogenesis, and increases gliosis, which lead to epileptogenesis [13–15]. The innate and acquired immune systems are activated in patients with epilepsy. Analysis of brain samples from patients with refractory epilepsy demonstrated heightened expression of IL-1β and high-mobility group box 1 (HMGB1), along with their corresponding receptors, namely interleukin-1 receptor type I (IL-1RI) and TLR4, in both glia cells and neurons. This clarifies that patients with epilepsy experience activation of inflammatory mechanisms [8, 13].

### Targets and sources of cytokines and inflammatory mediators in epileptic tissue

Microglia and astrocytes as glial cells and non-neuronal parts of the central nervous system are the source of pro-inflammatory cytokines in epileptic tissue. In epileptogenesis, glial cells control inflammatory and immunological responses, it causes a rapid inflammatory response in epileptic seizures caused by chemo seizures or electrical stimulation. Within the first 30 minutes of an epileptic seizure, activated astrocytes and microglia trigger heightened levels of pro-inflammatory cytokines, such as IL-1β, IL-6, and TNF-α. This process initiates an inflammatory cascade that involves neurons and BBB endothelial cells, ultimately leading to the activation of the acquired immune system. This cascade includes the activation of NF-κB, COX-2, complement system, chemokines, and acute phase proteins. These cytokines have the potential to influence susceptibility to stimuli that trigger epileptic seizures, as well as contribute to the increased IL-1β levels resulting from epileptic convulsions. The initiation of this signaling cascade exacerbates epileptic seizures. The conversion of IL-1β into its active form is facilitated by the enzyme IL-1β converting enzyme (ICE/caspase-1). Considering this, exploring ICE/caspase-1 inhibitors as potential targets for antiseizure medications holds promise [4, 8, 12].

Rapid HMGB1 release from neurons, microglia, and astrocytes after pro-convulsant-induced brain injury was also shown to significantly influence the onset of brain inflammation and lower the threshold for epileptic seizures. Toll like receptor (TLR) signaling also plays a significant role in astrocytes and neurons. Injured or stressed cells emit danger signals to warn the surrounding environment of impending or ongoing damage, including in the HMGB1. The innate immune response and accompanying inflammatory pathways are activated in the tissue due to the interaction between HMGB1 and its cognate TLR4 [4, 8, 12]

### The role of cytokines on neuronal excitability

Inflammatory cytokines play a role in changing neuronal excitability, producing toxic mediators, and making the BBB more impermeable. The proconvulsant effects are produced by NMDA receptors and IL-1β. Specifically, IL-1β can phosphorylate the NR2B subunit of the NMDA receptor via IL-1RI-dependent activation of Src kinases and neuronal sphingomyelinases. This phosphorylation amplifies NMDA-mediated calcium ion influx into neurons, thereby inducing neuronal hyperexcitability. Activated astrocytes release glutamate, which serves as the primary source, initiating a gradual influx of ions into neurons. This process involves NMDA receptors that incorporate the NR2B subunit. This influx of ions has a direct impact on excitatory events, exerting a substantial influence on neural synchronization and potentially giving rise to action potentials within neurons.

An essential biological mechanism that intensifies epileptic seizures involves the protein IL-1β. Furthermore, IL-1β has the capacity to reduce glutamate absorption by astrocytes, elevate glutamate release from glial cells through TNF-α, and induce hyperexcitability. The presence of IL-1β also leads to an inhibition of glutamate uptake by nitric oxide. Similar to IL-1β, TNF-α might boost the expression of AMPA receptors, facilitating the entry of calcium ions into neurons, while concurrently diminishing the expression of GABA receptors, thereby weakening the potency of inhibitory synapses. The signaling cascade mechanism governing glutamate exocytosis from astrocytes involves the participation of tumor necrosis factor (TNF-α). This process engages tumor necrosis factor receptor 1 (TNFR1) activation within astrocytes, thereby amplifying the signaling cascade responsible for glutamate release. This mechanism operates through the stimulation of CXCR4-dependent signaling in both astrocytes and microglia. Furthermore, it works synergistically to facilitate the release of TNF-α [4, 8, 12].

Inflammatory cytokines can lead to neuronal death through apoptosis, operating independently from excitotoxicity. This effect is potentially facilitated by the release of neurotoxic mediators. Additionally, glutamatergic excitotoxicity, which involves AMPA and NMDA receptors, can also contribute to neuronal demise. Inflammatory responses can also change the permeability of BBB. When BBB is damaged, astrocytes take up serum albumin. Within astrocytes, this albumin binds to TGF-β receptors, initiating a sequence of events that culminate in neuronal hyperexcitability and epileptiform activity. The impairment of the BBB leads to increased abnormal neuronal depolarization processes. The breach in the BBB allows leukocytes to enter the brain parenchyma and proteins to leak from the serum. These exogenous inflammatory mediators collectively reduce the threshold for epileptic seizures. This scenario underscores the significant impact of inflammatory processes on epileptogenesis within the brain [4, 8, 12]. The pathophysiology of epileptic seizures involves the adherence of inflammatory cells. After a pilocarpine hydrochloride-induced epileptic seizure, there is an increase in the production of vascular cell adhesion molecules (VCAM) and an increase in leukocyte adherence to endothelial cells in cerebral blood arteries, which is mediated by leukocyte mucin P-selectin glycoprotein ligand-1 and leukocyte integrins. Consequently, escalated leukocyte extravasation, brain inflammation, BBB leakage, increased neuronal excitation, and ultimately, the onset of epileptogenesis ensue [4]

Peripheral inflammation has been correlated with the stimulation of gene transcription through NF-κB, an unconventional intracellular signaling pathway activated by pro-inflammatory mediators within epileptogenic tissue. The recognition of this distinct mechanism provides insights into the genesis of epileptic seizures driven by neuronal hyperexcitability. This pathway regulates a subset of interactions between neuronal and glial elements that contribute to the reduction in the threshold for epileptic seizures [8, 16]. When pro-inflammatory molecules activate other kinase families, such as mitogen-activated protein kinase (MAPK), protein kinase A (PKA), and protein kinase C (PKC), voltage-dependent calcium, sodium, and potassium ion channels undergo rapid post-translational changes that have a significant effect on neuronal excitability. Increased extracellular glutamate levels are caused by IL-1 promoting glutamate release by glial cells and decrease glutamate absorption by astrocytes through the generation of TNF-α. Furthermore, the amplified glutamatergic transmission, a consequence of IL-1β produced by both neurons and astrocytes, yields pro-convulsant effects. Recent findings emphasize the critical role of astrocytic glutamate release in shaping the initiation and severity of epileptic convulsions. Additionally, IL-1 impedes inhibitory transmission by blocking GABA-mediated chloride ion influx. The presence of inflammatory compounds in the brain could also incite enduring transcriptional processes, thereby activating genes integral to the plasticity mechanisms underlying epileptogenesis [8, 16]

### The role of P2X7 receptors in epilepsy

The P2X7 receptor is a cation-permeable ATP-gated non-selective ionotropic receptor expressed only by neurons and glia cells in the brain. Neuronal excitability in the hippocampus was shown to be modulated by activation of this receptor, which is also linked to microglia activation and neuro-inflammatory reactions. These receptors play a role in the neurological system disorders, such as epilepsy. P2X receptors are ligand-gated ion channels that ATP activates, and activating these receptors transfers signals between neurons and glia cells. Potassium ion concentration in the extracellular space rises during an epileptic episode. The Pannexin1 (Panx1) channels of hippocampal neurons and astrocytes activate in response to this alteration, releasing ATP. Neuronal hyperactivity brought on by the activation of ATP receptors leads to the onset of an epileptic seizure. Adenosine, as opposed to ATP, exhibits anti-epileptic action via P1A1 receptors. These purinergic receptors are inhibited from releasing glutamate when they are activated. Several P2X family receptors, including P2X2, P2X4, and P2X7, are damaged in the hippocampus during status epilepticus (SE). Following SE, a reduction in P2X2 receptor expression was also observed. In contrast, P2X7 receptor expression was higher in glial and neuronal injury regions. After SE, neuronal death is reduced by a lack of P2X4 receptors [12].

## The structural, molecular, and functional alterations of BBB in epileptogenesis and epilepsy

BBB, which is impermeable to a wide range of chemicals, toxins, and cells due to the presence of non-fenestrated endothelial cells forming inter-endothelial tight connections, serves as a protective shield for the central nervous system, preventing any immune system reactions. The BBB is composed of both chemical and structural barriers. It comprises neovascular units such as the basement membrane, endothelial cells that line the blood arteries, neurons, astrocyte processes, tight junctions, and pericytes. These components collectively form the structural barrier of the BBB. Nectrin 1, functioning as a laminin-like protein responsible for regulating glial cell motility, is secreted by endothelial cells in response to signals from astrocytes, which concurrently produce the sonic hedgehog protein. In a concerted effort to fortify the BBB and diminish the infiltration of leukocytes, netrin 1 plays a crucial role in enhancing endothelial junctional proteins. These include occludins, claudins, junctional adhesion molecules (JAMs), and endothelial selective adhesion molecules (ESAM). This ensemble of proteins collectively widens the intercellular gaps, constituting the foundational elements of tight junctions and intercellular barriers. Members of the immunoglobulin superfamily, such as ESAM and JAMs, play a pivotal role in both the formation and upkeep of tight junctions. The basement membrane, constituting a foundational structure, embeds components like type 4 collagen, fibrillin, laminin, fibronectin, extracellular matrix proteins, and parasite matrix proteins, which collectively give rise to the basal capillaries or basement membrane. Within the brain capillaries, tight junctions are safeguarded by efflux transporters, forming a chemical barrier. These transporters, operating at the junctions between endothelial cells, necessitate the presence of ATP. Prominent among these are P-gp and breast cancer resistance protein (BCRP), functioning as flux transporters. Their active mechanism involves pumping potentially harmful lipophilic compounds back into the bloodstream, thereby restricting the assimilation of certain medicinal substances into the brain parenchyma. Furthermore, the chemical barrier of the BBB derives additional strength from the drug-metabolizing enzyme cytochrome P450 (CYP450), which is localized within the endothelial cells of brain capillaries. Studies by Rana & Musto [13] and Löscher & Friedman [17] have underscored these aspects. During epileptic seizures, the release of glutamate, facilitated by NMDA and COX-2 receptors, is accountable for the escalation in P-gp activity [12].

Seizures or the processes underlying them have the potential to impact the BBB, brain capillaries, and the neovascular unit in diverse ways. This can encompass thickening of the basement membrane, alterations in the constituent endothelial cells of the BBB, modifications in the neurons comprising the neovascular unit, and changes in the astrocyte processes forming the glia limitans—a secondary barrier. Epileptogenic brain injury can lead to the leakage of blood albumin and immune cells into the brain parenchyma. Notably, the escalated expression of efflux transporters such as P-gp and BCRP on the apical surface of endothelial cells, along with heightened activity of the enzyme CYP450 on these cells, has been associated with BBB damage. Concurrently, the presence of persistently activated microglia clusters may contribute to the disruption of the BBB. Furthermore, post brain injury, astrocytes undergo alterations in both morphology and function. These changes can collectively lead to a complex interplay of factors influencing the integrity and functionality of the BBB and related systems [17].

The mechanism by which epileptic convulsions impact the BBB remains uncertain. Observations in epilepsy patients and experimental animal models suggest that BBB failure can occur before, during, or after an epileptic seizure. Epileptic convulsions induce temporary changes in both the structural and functional aspects of the BBB. Within 30 min of an epileptic episode, there is a rapid increase in permeability that persists for hours. Elevated levels of glutamate during epileptic episodes disrupt the BBB. The expression and activity of MMP-2 and MMP-9 at the BBB also rise during seizures, contributing to BBB dysfunction. These matrix metalloproteinases influence the integrity of the BBB by altering the extracellular matrix surrounding brain capillaries and degrading the tight junction proteins that line the endothelium. The extracellular matrix, in turn, affects processes like AMPA receptor mobilization, paired-pulse depression, L-type voltage-dependent calcium channel activity, and the long-term potentiation (LTP) process. Changes in the expression of hyaluronic acid, a crucial component of the neuronal extracellular matrix, can lead to dysregulation of calcium ions and neuronal hyperexcitability. Inducing epileptic seizures can also permit ions to enter the parasite, thereby modifying its function and the constituents of the BBB both in vitro and in vivo. These defense system breaches, causing epileptic seizures, can establish a positive feedback loop. In this loop, seizures resulting from these breaches lead to a continuation of epileptic episodes, ultimately culminating in epilepsy. The breach of the cerebral blood barrier defense can trigger epileptic episodes and contribute to the development of epilepsy [6, 14, 17].

The BBB serves as a crucial barrier to maintain the peripheral immune cells in a healthy state, preventing their entry into the central nervous system. However, when breached, the BBB can allow both innate and acquired peripheral immune cells, including monocytes, neutrophils, B cells, and T cells, to infiltrate the central nervous system. These immune cells then carry out specific functions that could be either neuroprotective, neurotoxic, or even a combination of both. A significant facet of neuro-inflammation involves the entrapment of these immune cells within the central nervous system through the BBB. This orchestrated process hinges on adhesion interactions between lymphocytes and capillary endothelium. When immune cells come into contact with the activated endothelium, selectins facilitate loose binding, causing the leukocytes to roll along the endothelial surface. Additionally, chemokines present on the luminal surface of the endothelium trigger the activation of leukocytes, leading to the engagement of leukocyte integrins and subsequent halting of leukocyte movement on the endothelial surface. Once the appropriate adhesion occurs, immune cells can traverse into the perivascular space through either of two transmigration processes: the trans-cellular route, where they navigate through the endothelial cell body, or the paracellular route, utilizing the endothelial cleft. Upon infiltrating the perivascular space, these immune cells interact with resident perivascular immune cells before being guided by chemoattractants like chemokines and cytokines toward the brain parenchyma. Employing an enzyme-mediated mechanism, these infiltrating lymphocytes breach the glia limitans and access the brain parenchyma. Once within the brain parenchyma, they become stimulated to produce cytokines, contributing to the cascade of immune responses and inflammation. The BBB serves as a sentinel that regulates the entry of peripheral immune cells into the central nervous system. Breaching the BBB leads to a series of orchestrated events involving adhesion, activation, and migration of immune cells, ultimately culminating in their interaction with brain parenchymal cells and the release of cytokines [17].

Albumin extravasation plays a significant role in both epileptogenesis and the migration of immune cells across a compromised BBB. The initiation of acute epileptic seizures can be attributed to BBB impairment, and the progression from epileptic episodes to epilepsy is facilitated by the osmotic disruption of the BBB. Once extravasated, albumin enters the brain parenchyma and binds to various cellular components, including neuronal cells, astrocytes, and microglia. This binding triggers a sequence of events, including the entry of albumin into astrocytes through the TGF-β receptors. Consequently, the expression of critical components such as the Kir 4.1 channel, the water channel aquaporin 4 (AQP4), and glutamate transporters is reduced. This alteration results in a decreased ratio of extracellular potassium to glutamate, creating an environment that facilitates NMDA receptors in triggering epileptiform activity and amplifying neuronal hyperexcitability. The impact of TGF-β signaling extends further, encompassing changes in the extracellular matrix, excitatory synaptogenesis, pathological plasticity, and inflammation-related transcriptional alterations. These cumulative effects collectively contribute to the lowering of the threshold for epileptic seizures during the process of epileptogenesis. In essence, albumin extravasation sets in motion a complex cascade of events that involve multiple cellular components and signaling pathways. This interplay ultimately heightens neuronal excitability and paves the way for epileptogenesis and the progression towards epilepsy [17]. Albumin binding to TGF-RII receptors on astrocytes triggers the activation of the TGF-β signaling pathway, leading to the production of TGF-β. This, in turn, activates astrocytes, causing a disruption in the balance between cellular potassium and glutamate levels. Additionally, the ALK5/TGF-β pathway is responsible for inducing excitatory synaptogenesis [13]. In models of epileptogenesis, augmenting TGF-β signaling has been shown to have a preventive effect on the development of epilepsy. Furthermore, another study established a connection between compromised BBBs and irregular neuronal activity [17].

Damage to the BBB triggers gliosis and activation of astrocytes. After inducing SE, astrocytes positive for glial fibrillary acidic protein (GFAP) become activated within a span of 24 to 48 h. Reactive gliosis becomes evident around three to four months following the initial insult. The inability of these reactive astrocytes to effectively manage extracellular glutamate leads to hyperexcitability and neuronal damage. Furthermore, these activated astrocytes release C-C motif chemokine ligands 2, 3, and 5, fostering interactions with other inflammatory cells and giving rise to the production of pro-inflammatory cytokines such as IL-1β, IL-6, and TNF-α. This cascade contributes to heightened neuronal excitability, the onset of seizures, cell death, and the propagation of neuro-inflammation [13].

## The role of mTOR pathway in epileptic seizure and epilepsy

The excessive activation of the mTOR signaling pathway directly influences the progression of epileptogenesis and neuronal excitability. This process involves the phosphorylation of molecules within this pathway. Additionally, the mTOR pathway plays a role in governing the expression of glutamate receptors. Recent research further underscores the potential for disrupted neural signaling, as mTOR contributes to both synapse formation and the mechanisms underlying plasticity [12]. Moreover, mTOR exerts control over the expression of diverse ion channels and neurotransmitter receptors, as well as influencing synaptic plasticity, neuronal morphology, and synaptic transmission [18].

In prevalent causes of hereditary epilepsy such as focal cortical dysplasia (FCD) and tuberous sclerosis complex (TSC), the involvement of the mTOR pathway in epileptogenesis is notably evident. Beyond these conditions, the mTOR pathway plays a role in the development of both inherited and acquired epilepsies, encompassing conditions like Lafora disease (LD), temporal lobe epilepsy, brain injuries, and experimental epileptic states induced by chemically triggered convulsions. Additionally, the mTOR pathway is implicated in systemic lupus erythematosus, an inflammatory disorder that stands as a prominent contributor to epileptic seizures [19]. Genetic mutations impacting various components of the mTOR pathway, such as *TSC1* and *TSC2*, STE20-related kinase adaptor-α (*STRADA*), phosphatase and tensin homologue (*PTEN*), AKT isoform predominant in the brain (*AKT3*), phosphatidylinositol-4,5-bisphosphate 3-kinase-α (*PIK3CA*), and MTOR itself, result in the hyperactivity of the mTOR pathway. This hyperactivity, in turn, triggers the initiation of epilepsy syndromes [20–24]. Conversely, the inhibition of mTOR can forestall the onset of epilepsy. The modifications in autophagy driven by mTOR are interconnected with the process of epileptogenesis. Following mTOR hyperactivation, autophagy experiences suppression, and the commencement of spontaneous epileptic seizures is associated with the breakdown of autophagy. Autophagy governs diverse processes including axon guidance, synapse development, dendritic spine architecture and pruning, vesicular release, and synaptic plasticity. It also exercises control over the proliferation and migration of both interneuronal and neuronal cortical progenitors.

Evidence points towards mTOR-mediated autophagy failure giving rise to aberrations in the clustering of GABAA receptors at synapses, thereby leading to an imbalance between excitation and inhibition—an essential factor in epileptogenesis. Perturbations with irregular activity of  the dopamine system additionally influence the functioning of mTOR-mediated autophagy, contributing to epileptogenesis. This intricate interplay arises from bidirectional communication and shared biochemical pathways, involving cross-reactions between the immune and neurological systems. The connection between mTOR and cell-clearing mechanisms responsible for regulating the metabolic processes of lymphocytes and other immune cells, as well as the antigen-processing capabilities of antigen-presenting cells (APCs) in both the central and peripheral nervous systems, further bolsters this notion. Changes in mTOR-associated cell-clearing processes can disrupt and misalign the communication between the immune and neurological systems [19]. In parallel with its impact on autophagy, epilepsy also exerts effects on the ubiquitin-proteasome system (UPS), a regulatory network governing neuronal excitability, synapse plasticity, and neuro-inflammation/immunity. Disturbances in the UPS within the context of epilepsy are associated with mTOR hyperactivation, akin to the phenomenon observed with autophagy disruption. Recent investigations have elucidated the role of mTOR in orchestrating the convergence of autophagy and UPS morphological pathways, as well as their collective cell-clearing mechanisms. These findings underscore that mTOR-mediated autophagy and UPS activities collectively contribute to epilepsy by influencing brain plasticity and promoting neuroprotection against excitotoxicity. This interplay sheds light on the multifaceted mechanisms through which epilepsy shapes neurological processes and underscores the significance of mTOR regulation in these contexts [19].

**Fig. 2:**
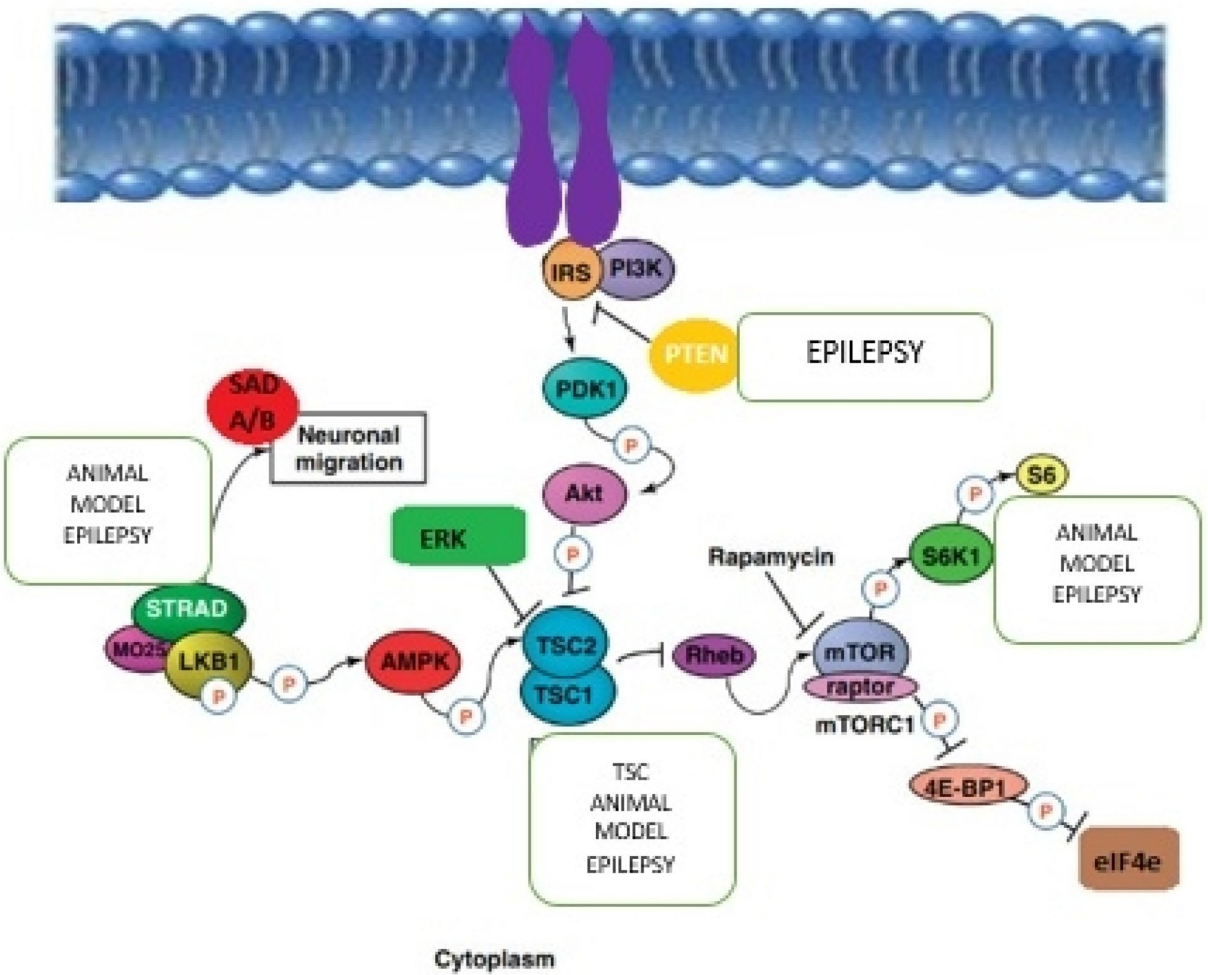
The role of mTOR pathway in epileptogenesis. Dysmorphic neurons and balloon cells, which lack dendrites or axons, undergo alterations in neurotransmitter receptor subunit expression and uptake site distribution in conditions like TSC, FCD, and hemimegalencephaly (HME). Notably, changes are prominent in GluR, NMDAR, and mGluR subtypes. The direct causation of these alterations—whether they arise from heightened mTOR signaling, tissue cytoarchitecture changes, or adaptations stemming from recurrent seizures—remains uncertain. The accompanying diagram illustrates various locations where dysregulation of mTORC1 (influenced by both phosphoactivation and phosphoinhibition effects) is implicated in human neurological disorders collectively referred to as “mTORopathies,” which encompass epilepsy. Components such as PTEN, STRADA, TSC1/TSC2, and S6K1 might play roles in this context. Experimental animal models involving these proteins also exhibit compromised seizure susceptibility and/or deficits in social and behavioral functions. Rapamycin and other mTOR inhibitors emerge as primary potential therapeutic agents for modulating mTORC1 signaling. These interventions hold promise for regulating the mTOR pathway, offering prospects for addressing the underlying mechanisms of mTOR-related neurological disorders, including epilepsy [21, 25]. Adapted from Crino [21]

Autophagy operates at the interface of the GABA, dopamine, and glutamate systems, contributing to the molecular mechanisms underlying epileptogenesis and the neuronal changes prompted by epileptic convulsions. In instances of autophagy failure, heightened p62 levels conceal the surface presentation of GABAA receptors, leading to a reduction in GABA signaling. Furthermore, the degradation of NMDAR and AMPAR glutamate receptors no longer takes place, resulting in irregular glutamate signaling and an influx of calcium ions. This disarray disrupts the delicate equilibrium between excitatory and inhibitory neurotransmission pivotal to epileptogenesis. On the dopaminergic front, the role of autophagy extends to curtailing dopamine release through the disintegration of synaptic vesicles at the dopamine terminal. In cases where autophagy falters, anomalous dopamine release ensues, accompanied by the stimulation of dopamine D1 receptors. This, in turn, exacerbates autophagy suppression through mTOR hyperactivation. This detrimental cycle leads to the accumulation of damaged cellular components, which combine with glutamate-associated excitotoxicity, ultimately culminating in neuronal demise [19] (Fig. 2).

## The role of apoptotic pathway in epileptic seizure and epilepsy

Beyond its pivotal role in neuronal development, differentiation, and synaptic plasticity [26, 27], glutamate receptor-mediated excitotoxicity is also recognized as a major mechanism contributing to cell death in various disorders of the central nervous system (CNS). These include conditions like brain injury, neurodegenerative diseases, and epilepsy [28]. Elevated levels of glutamate receptor activity have the potential to induce cell damage and subsequent death through mechanisms involving apoptosis or autophagy. As a consequence of heightened glutamate receptor activity, the expression of pro-apoptotic proteins such as p53 is triggered [29–31]. This activation of pro-apoptotic proteins is often prompted by immediate excitotoxic injury [32, 33].

Excessive activation of glutamatergic receptors or their analogs leads to a phenomenon known as excitotoxicity, which ultimately results in cell death. When these receptors experience an overstimulation, an excessive influx of calcium ions (Ca^2+^) occurs, with a significant portion becoming sequestered within mitochondria. Consequently, cellular metabolism becomes impaired, giving rise to the generation of free radicals. This excessive calcium influx also triggers the activation of enzymes like proteases, phospholipases, endonucleases, and nitric oxide synthase. Furthermore, the process inhibits protein synthesis, exacerbating the cascade of detrimental events within the affected cells [34]. Disruption of calcium homeostasis leads to an accumulation of calcium within the mitochondrial matrix. This accumulation has the effect of depolarizing the mitochondrial membrane through the activation of mitochondrial transition pores, which normally function to redistribute calcium back to the cytosol. This process unfolds in two primary steps. Initially, the elevation in positive ion concentration within the mitochondrial matrix diminishes the chemo-osmotic potential across the membrane. This reduction in potential subsequently slows down the rate at which ATP synthesis occurs. In the second step, irreversible damage takes place at the mitochondrial membrane. This disruption in calcium regulation and the ensuing impact on mitochondrial function represent critical components of the excitotoxic cascade [35, 36]**.**

**Fig. 3:**
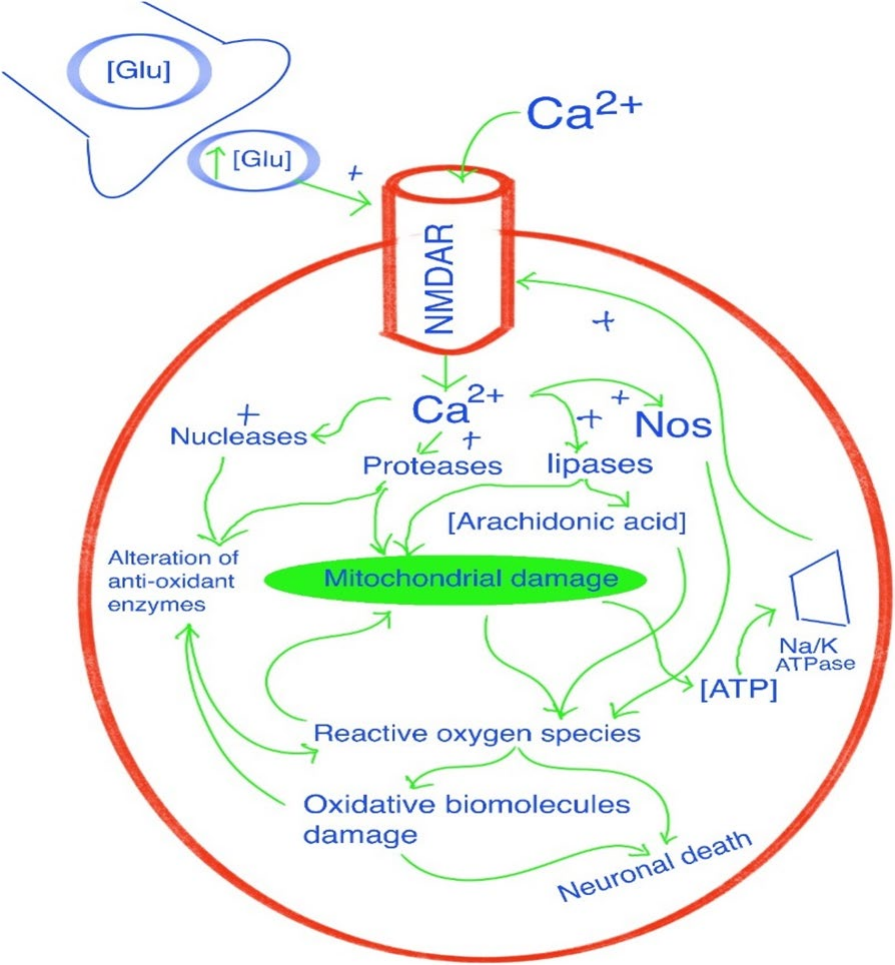
The mechanism of excitotoxicity. The sustained activation of N-methyl-D aspartate receptors (NMDAR) due to excessively high concentrations of glutamate (Glu) results in a substantial influx of calcium into the cell. This influx, in turn, triggers the activation of lytic enzymes and nitric oxide synthase (NOS). Concurrently, heightened levels of arachidonic acid and mitochondrial impairment foster the generation of reactive oxygen species, initiating a cascade of biomolecular damage. Ultimately, these processes lead to the activation of apoptotic death programs within cells. The impact of energy shortages compounds the degenerative process. Energy deficits prolong membrane depolarization by impairing the energy supply to the sodium/potassium pump (Na/K ATPase). This, in turn, maintains NMDAR in an active state, rendering the cell more susceptible to the typical glutamatergic inputs from the cerebral cortex. Adapted from Lorigados et al [38]

The complexity of neuronal cell death associated with neurodegeneration is not mitigated by neuronal cell death triggered by epileptic seizures. However, the precise nature of neuronal cell demise, whether through necrosis or apoptosis, remains a subject of debate. While necrosis is frequently cited as the principal cause of neuronal cell death following epileptic seizures and is widely considered the predominant mechanism, the discussion persists. Biochemical investigations have unveiled the involvement of the B-cell lymphoma 2 (Bcl-2) family and caspases in the cell death process during epileptic seizures. Consequently, the potential contribution of apoptosis cannot be dismissed. Indicative of apoptosis, DNA fragmentation and activation of endonucleases have been identified in the cell death program. Additionally, there is evidence of p53 accumulation in the nucleus in tandem with increasing concentrations of cell death receptors and ligands. These indications point to the involvement of apoptotic pathways in contributing to cell death [37, 38] (Fig. 3).

The *p53* gene emerges as the initial apoptotic regulator disrupted by an epileptic episode. Subsequent to an epileptic seizure, the process involves the binding of p53 to DNA. This is accompanied by heightened expression of Bcl-2-associated X (Bax) protein, which in turn leads to increased levels of both mRNA and protein. Protection against excitotoxicity induced by kainic acid was observed when inhibitors of p53 production were administered. Studies involving p53-deficient mice’s neurons indicated heightened resilience to apoptosis induced by excitotoxicity and epileptic seizures. Given the multifaceted roles of p53, it remains unclear precisely how p53 is altered in epileptic seizures that trigger cell death. However, the prevailing evidence predominantly points toward caspases, Bcl-2, and p53 as integral components of the pathways underlying neuronal cell death induced by epileptic seizures [38].

In mammals, the regulation of the apoptotic process revolves around two principal gene families: caspases and the Bcl-2 family of proteins. The caspase family, composed of caspases 2, 8, 9, and 10 as initiators, and caspases 3, 6, and 7 as executors, serves pivotal roles within the apoptotic pathway. On the other hand, the Bcl-2 family of proteins exerts crucial control over whether a cell will survive or undergo apoptosis. This family can be categorized into three distinct types of proteins, each of which contributes uniquely to the apoptotic process. The first group encompasses anti-apoptotic proteins featuring four Bcl-2 homology (BH) domains: BH1-4. The second category, effector proteins Bax and Bak include BH1-4 domains and. The third set is marked by the presence of the BH3-only domain (Bid, Bad, Bim, Bik, etc). Among these, the BH3-only proteins and effector proteins of the Bcl-2 BH family act as pro-apoptotic factors. The anti-apoptotic Bcl-2 family proteins such as Bcl-2 and Bcl-XL bind to the pro-apoptotic protein Bax/Bak, inhibiting their capacity to form mitochondrial membrane pores. Conversely, pro-apoptotic proteins like Bid, Bad, Bim, and Bik detach Bax/Bak from the Bcl-2-Bax/Bak or Bcl-XL-Bax/Bak complex, thereby initiating Bax translocation to the mitochondrial membrane and facilitating pore formation. Upon release from the anti-apoptotic protein connection, Bax/Bak dimerizes and remains in the active mitochondrial membrane. Subsequently, the creation of mitochondrial pores leads to the release of crucial proteins like cytochrome C, pivotal for triggering apoptosis through the mitochondrial pathway. Alongside caspases and Bcl-2 family proteins, other prominent players, including p53, TNF, Fas ligand, and NF-κB, play significant roles in orchestrating the cell death pathway [4].

**Fig. 4:**
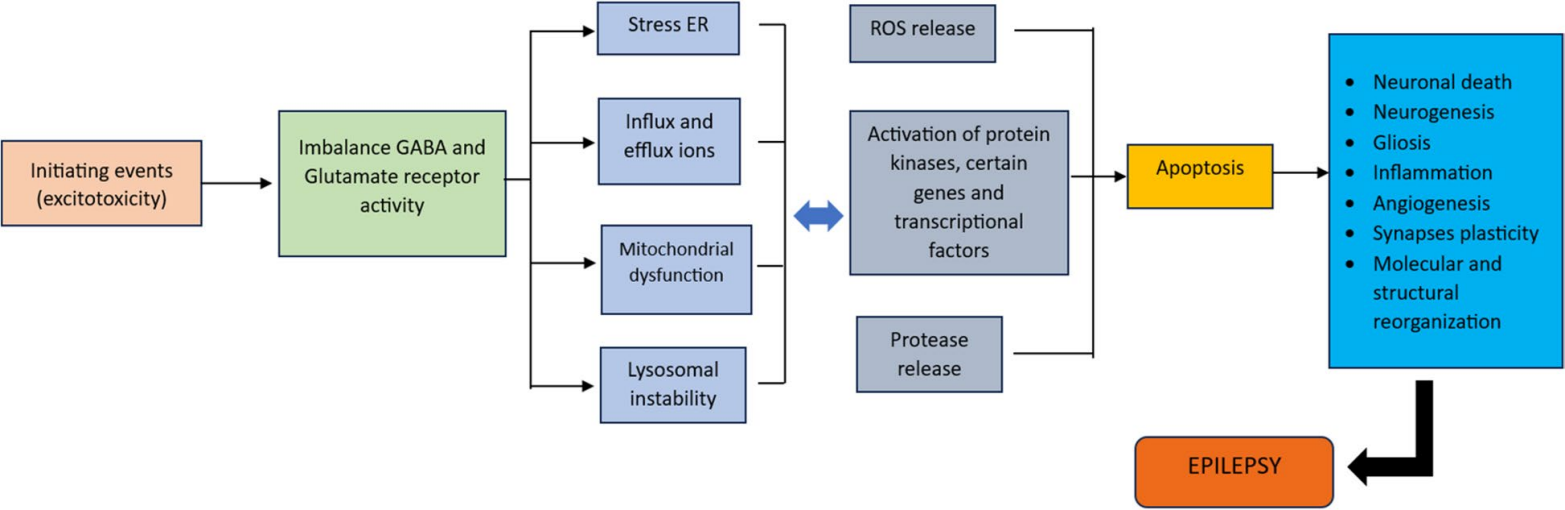
The mechanism scheme of excitotoxicity induces epilepsy. Adapted from Lorigados et al [38]. ER: endoplasmic Reticulum, ROS: reactive okxidative stress

The process of excitotoxicity involves the hyperactivation of glutamatergic receptors and their analogs, leading to cell death (Fig. 4). The excessive influx of calcium ions into the cell, as a result of this hyperactivation, accumulates within mitochondria. This accumulation triggers a chain of events including metabolic dysfunction, the generation of free radicals, activation of enzymes like proteases, phospholipases, endonucleases, and nitric oxide synthase, as well as the inhibition of protein synthesis. Additionally, disruptions in calcium ion homeostasis stem from malfunctions in the calcium ion pump, sodium and calcium ion exchange mechanism, and calcium-balancing proteins. When this delicate balance is perturbed, surplus calcium ions amass in the mitochondrial matrix. There are two primary mechanisms through which this buildup occurs. Firstly, an elevated concentration of positive ions within the mitochondrial matrix reduces the chemo-osmotic potential across the membrane, slowing down the rate of ATP synthesis. Secondly, the activation of the mitochondrial transition pore aims to transport calcium ions back to the cytosol. However, if the returned calcium ions are not effectively cleared, it results in irreversible membrane depolarization. The heightened concentration of calcium ions in the mitochondrial matrix further fosters the generation of free radicals. This, in turn, leads to lipid membrane peroxidation, nitric oxide production, and the activation of enzymes involved in the breakdown of proteins, phospholipids, and nucleic acids. Notably, nitric oxide can also act as a retrograde messenger, amplifying glutamate release from the terminal presynapse, thereby exacerbating its excitotoxic effects [38].

## Ionic environment modifications causing epileptic seizures and epilepsy

While certain forms of epileptic seizures might originate in subcortical regions, the majority of such seizures stem from the cortical and hippocampal structures. Various factors, including the site of disruption, the timing and duration of the electrical bursts, and the associated clinical manifestations, collectively contribute to the complexity of epileptic seizures. Beyond instigating abnormal activity within individual neurons, alterations in excitability, intrinsic to epileptogenesis, encompass networks of hyperexcitable cells. These cells function cohesively, demonstrating a high degree of coordination via both functional and potentially dysfunctional pathways [39].

Upon membrane depolarization, voltage-gated sodium channels are activated, leading to a pivotal shift in the action potential phase. Concurrently, various voltage-gated potassium channels, integral to the repolarization phase, initiate their activity. Following their activation, sodium ion channels transition into an inactive state due to the ball-and-chain mechanism, which closes the channel pore, effectively halting further sodium ion passage. Notably, as the action potential reaches its zenith, the majority of sodium ion channels are already inactive, with an exception being the persistent sodium channels (*I*_NaP_) that remain open. Even this subtle continuation of sodium ion conduction by *I*_NaP_ channels suffices to propel the membrane past the action potential threshold. This mechanism forms the basis of the intrinsic electrical impulse observed in layer V pyramidal neurons. Consequently, this inherent electrical discharge extends throughout axon branches, fostering synaptic connections with proximate neurons and preserving their synchronized activity [39].

Ion channels are integral proteins that form pores within the lipid membrane of a cell. These pores facilitate the selective passage of specific ions, thereby contributing to the maintain the negatively charged resting membrane potential of cells. These membrane proteins, termed hetero-oligomeric ion channels, consist of two to six subunits. Each subunit features transmembrane segments and differing domain counts, culminating in the creation of diverse channel subtypes. The assembly of these subunits generates a range of channels, each equipped with distinct functionalities. Ion channels are classified as either voltage-gated or ligand-gated, contingent upon how they respond to alterations in the membrane potential. Ligands such as GABA and the neurotransmitter acetylcholine can activate ion channels, serving as prime examples. Cation channels play a pivotal role in initiating the action potential, thus contributing to neuronal excitability. Conversely, anion channels are instrumental in executing inhibitory processes that counteract neuronal stimulation. Disturbances in ion charge equilibrium resulting from anomalies in anion or cation channel function, known as channelopathies, have the potential to trigger epileptogenesis [4, 39, 40].

### Sodium ion channels

The movement of sodium ions through the inactivation of fast channel propels the action potential phase upward. This phenomenon is intertwined with the inactivation of the slower sodium ion channel component, a process pivotal in governing membrane excitability. The presence of toxins that hinder the rapid inactivation of sodium ion channels can additionally amplify the inactivation of the slow component. This dynamic shift leads pyramidal neurons in the neocortex to transition from regular spiking to burst firing. This alteration can be epileptogenic in two distinct ways. Firstly, it can trigger paroxysmal depolarizing shifts (PDS) inducing electrical bursts within the affected neurons. Secondly, it can enhance synchronization stemming from intrinsic electrical bursts in the pyramidal neurons of the neocortex’s layer V. As a consequence, a substantial cohort of neurons becomes engaged, thereby laying the foundation for an epileptogenic process to take root [39, 40].

### Potassium ion channels

Calcium ion influx triggers the activation of potassium channels, playing a pivotal role in the subsequent repolarization of the cell membrane following excitation. In fact, the calcium ion-dependent flow of potassium ions maintains its distinct contribution to post-action potential hyperpolarization in cells intrinsically stimulated, such as pyramidal neurons within the CA area. This potassium ion movement encompasses the M current, named due to its inhibition by muscarinic acetylcholine receptor activation—an integral mechanism for regulating neuronal excitability. The M current holds significance in governing membrane excitability below the threshold and the ability to respond to synaptic input. Its activation relies on a slow conductance that occurs between -60 and -20 mV. Blocking the M current by muscarinic receptor binding induces a shift in the neuronal membrane towards greater depolarization. Consequently, this alteration can lead to the occurrence of repetitive action potentials interspersed with periods of burst activity. In the context of benign neonatal familial convulsions, genetic factors linked to M current deficiency play a pathogenetic role. This M current is governed by two potassium ion channel subunits, namely KCNQ2 and KCNQ3, and mutations within the genes encoding these subunits—located at chromosome positions 20q13 and 8q24, respectively—contribute to M current disorders that are associated with the phenotype of benign neonatal familial convulsions [39, 40].

### Calcium ion channels

Calcium ion channels play a crucial role in transient and persistent cell membrane depolarization, driven by their threshold and kinetic activation properties. However, understanding the involvement of calcium ion flux in epileptogenesis poses several challenges. Firstly, due to its essential role in protein synthesis and metabolism, the majority of calcium ions are stored within the endoplasmic reticulum. Manipulating calcium ion concentrations artificially, as often done in experimental setups, can trigger the release of stored calcium ions from their compartments, thereby introducing complex effects on cell excitability that are further compounded by metabolic influences. Secondly, when assessing the impact of calcium ion concentration on membrane excitability, it’s important to consider its role in mediating vesicle fusion in nerve terminals. This fusion process is vital for neurotransmitter release and synaptic function, adding another layer of complexity to the relationship between calcium ions and excitability. Thirdly, intentional reduction of calcium ion concentration can disrupt various types of calcium-dependent potassium currents that contribute to cell excitability. This disruption can lead to indirect and unpredictable outcomes that directly influence calcium ion movement through the cell membrane. This interconnectedness makes it challenging to isolate the precise effects of altered calcium ion concentrations on cellular excitability. Comprehending the role of calcium ion flux in epileptogenesis is intricate due to its multifaceted functions. Manipulating calcium ion concentrations can trigger cascading effects on cell excitability, neurotransmission, and potassium currents, making it challenging to draw definitive conclusions about its contribution to the epileptogenic process [39, 40].

### Excitatory amino acid receptors

In the cerebral cortex, the two primary excitatory amino acid neurotransmitters are glutamate and aspartate. These neurotransmitters exert their effects through distinct receptor subtypes. The specific level of ion permeability and the corresponding rates of ion flow are governed by the composition of their respective subunits [41, 42]. Two distinct types of receptors exist: NMDA and AMPA (with kainate and quisqualate subtypes). The ionophores associated with these receptor types allow the permeation of sodium ions, while the NMDA receptor type also permits calcium ion entry. NMDA receptors are blocked by magnesium ions, which remains dependent on the membrane’s polarization state. To activate this channel, sufficient depolarization of the membrane must first occur to remove the magnesium blockage. Glutamate binds to metabotropic receptors as well, which serve as second messengers in addition to ionotropic receptors. The processes involving sodium and calcium ions in the AMPA and NMDA components of excitatory postsynaptic potentials (EPSPs) are potent enough to sustain membrane depolarization, significantly extending and amplifying EPSPs. Anomalously formed synaptic connections, resulting from sprouting mossy fibers, can lead to the creation of additional excitatory feedback circuits. This feedback loop augments conductance facilitated by NMDA receptors [39, 43].

### GABA receptors

Chloride and potassium ionophores are attributed to the GABAA and GABAB subtypes, respectively, among the primary classes of ionotropic GABA receptors. While it has been proposed that the third receptor subtype, GABAC, may serve physiological functions, this remains uncertain. GABA binding to its receptors induces membrane hyperpolarization and generates inhibitory postsynaptic potentials (IPSPs), accomplished through the entry of chloride ions and the exit of potassium ions. GABA-mediated IPSPs have a notable ability to quell neuronal electrical bursts, primarily due to their connection with reduced input membrane resistance. Local inhibitory circuits comprised of Golgi type 2 GABAergic neurons regulate the main population of pyramidal neurons in the cerebral cortex. In individuals with refractory temporal lobe epilepsy, reduced GABAergic neuron density and impaired GABAergic circuit function have been observed in brain tissue. This underscores the role of GABA-inhibitory abnormalities in the progression of epilepsy [39].

### Acetylcholine receptors

Nicotinic acetylcholine receptors hold particular significance due to their presence in the central nervous system. These receptors form pentameric structures composed of three subunits: one subunit from the α group (α1 - α8) and another subunit from the β, γ, or δ group. Positively charged ions such as sodium, calcium, and potassium flow through the channel to achieve equilibrium. The channel is permeable to these ions without showing selectivity. The equilibrium potential of these ion channels can vary, although it typically hovers around -5 mV. Epileptic symptoms have been observed in individuals with mutations in the *CHRNA4* gene, responsible for encoding the receptor subunit 4, and the *CHRNB2* gene, responsible for encoding the nicotinic acetylcholine receptor 2 subunit. Investigating the precise impact of these mutations on neuronal hyperexcitability is crucial for a comprehensive understanding of their role [39, 40] (Table 1).

**Table 1:** The function of ion channel and receptor in normal and epileptic tissue [7]

Ion channels or receptor	Normal brain	Epileptic brain
Voltage-gated Na^+^ channles	Subthreshold EPSP, upstroke of the action potential	Repetitive action potential firing
Voltage-gated K^+^ channles	Action potential downstroke	Action potential repolarization
Voltage-gated Ca^+^ channles	Released transmitter, transports a depolarizing charge from the dendrite to the soma	A pathogenic intracellular process is activated by excessive transmitter release
Ca^2+^-dependent K^+^ channels	After hyperpolarization following action potential, sets refractory period	Limit repetitive firing
Non-NMDA receptor	Fast EPSP	Initiates paroxysmal depolarization shift
NMDA receptor	Prolong, slow EPSP	Maintain paroxysmal depolarization shift, Ca^2+^ activates pathologic process
GABA receptor	IPSP	Limit excitation
Na^+^ - K^+^ pump	Restore ionic equilibrium	Prevent K^+^ - induced depolarization

*EPSP* excitatory postsynaptic potential, *IPSP* inhibitory postsynaptic potentials

Recent advances in genetics and molecular biology have shown the connection between certain epilepsy disorders and mutations in genes responsible for coding ion channel proteins. These mutations can lead to a condition known as channelopathy, characterized by the malfunction or impairment of ion channels, resulting in heightened neuronal excitability [4, 6]. Various forms of familial epilepsy arise from genetic anomalies that disrupt the proper functioning of voltage-gated ion channels. These ion channels play a crucial role in regulating membrane potential, depolarization threshold, and synaptic transmission, all of which contribute to maintaining the normal excitability of cells. Mutations affecting these ion channels can lead to functional changes that result in neuronal hyperexcitability, ultimately triggering epileptic seizures [8]. Channelopathies have a substantial impact on epileptogenesis, particularly in patients with epilepsy, including those with idiopathic forms. Idiopathic epilepsy has been associated with mutations occurring in genes responsible for encoding various types of ion channels such as potassium, sodium, chloride, and calcium channels, as well as acetylcholine and GABA receptors. These mutations can lead to alterations in ion channel function due to changes in transcriptional and post-translational processes. Consequently, channelopathies have the potential to contribute to the underlying pathology of acquired epilepsy [4]. Mutations in key components of the inhibitory system can lead to the breakdown of normal inhibitory processes, thereby contributing to neuronal hyperexcitability in epilepsy. GABA, the principal inhibitory neurotransmitter, acts through ligand-gated chloride ion channels, which facilitate fast inhibitory synaptic transmission. Mutations in specific genes, such as the voltage-gated chloride channel (*CLCN2*), GABAA receptor subunit α1 (*GABRA1*), and GABAA receptor subunit γ2 (*GABRG2*), can disrupt the proper functioning of these components. These genetic alterations can result in the dysfunction of GABA conductance and chloride ion flux. As a consequence, the delicate balance between excitatory and inhibitory neurotransmission is disrupted, leading to an increased susceptibility to neuronal hyperexcitability, which is a hallmark of epilepsy. Understanding the impact of these mutations on inhibitory signaling pathways provides crucial insights into the underlying mechanisms of epilepsy [8, 44].

Mutations in genes responsible for encoding sodium channel proteins are believed to be a mechanism underlying certain types of inherited epilepsy. In humans, genes like *SCN1A*-*SCN5A* and *SCN8A*-*SCN11A* code for nine different subtypes of the alpha-subunit of voltage-gated sodium channels, specifically Nav1.1-Nav1.9. These mutations also impact epilepsy-related pathophysiology in the beta1 subunit of voltage-gated sodium channels. When mutations occur in the beta1 subunit, the affected sodium channels remain open for prolonged periods, allowing excessive sodium influx into the cell. Consequently, neurons become hyperexcitable and remain depolarized for extended durations. This leads to the release of large amounts of excitatory neurotransmitters, such as glutamate. As a result, the post-synaptic cells that interact with neighboring glutamatergic neurons experience excessive stimulation, leading to the production of calcium ions, which can be neurotoxic. Mutations in the genes encoding the alpha1 voltage-gated sodium channel subunit (*SCN1A*), which accelerates recovery from an inactive state, can also cause sustained depolarization due to prolonged sodium ion inflow and hyperexcitability. Hereditary epilepsy is linked to mutations in genes producing the voltage-gated sodium channel subunit beta1 (*SCN1B*) and *SCN2A*. Generalized epilepsy with febrile seizures involves mutations in sodium channel subunits *SCN1A* and *SCN2A*, as well as the GABAA receptor subunit *GABRG2*. Another form of generalized epilepsy caused by a single gene mutation is benign familial newborn convulsions. These genetic variations affecting sodium channels significantly contribute to the development of epilepsy by disrupting the normal balance of neuronal excitability and neurotransmission [8, 10, 12, 44, 45].

Potassium ion channels regulate neuronal excitability by allowing the flow of potassium ions to the extracellular space. This process helps control the resting membrane potential and facilitates rapid repolarization of the action potential. These ion channels consist of four alpha subunits that form the pores, alongside four beta subunits for regulation. Among the most significant subset of ion channels are the Kv channels (Kv1-Kv12), which are highly abundant in the central nervous system. Dysfunctions in Kv channels, including calcium-activated potassium ion channels, have been associated with epilepsy. Channelopathies related to epilepsy involve HCN and Kv7 channels. HCN and Kv7 channels, which facilitate cation transport, induce hyperpolarization through voltage-gated ion channels. HCN channels are prominently expressed in the hippocampus, cortex, and thalamus, contributing to localized and widespread epileptic seizures. Kv7 channels also play a role in modulating neuronal hyperexcitability. The HCN channel, however, performs a more intricate role. Genetic deletions in the HCN2 channel subtype do not result in an epilepsy phenotype, whereas deletions in the HCN subtype1 (HCN1) can accelerate epileptogenesis. The absence or inhibition of HCN1 channels increases neuronal hyperexcitability, seizure severity, and the risk of SE-related mortality. Membrane hyperpolarization activates HCN channels, inhibiting *I*_h_, a current that regulates neuronal excitability by controlling the resting membrane potential. Reduced *I*_h_ density due to decreased or lost channel expression can lead to neuronal hyperexcitability. Mutations in the *KCNQ* canal gene, responsible for Kv7 channels, are also associated with epilepsy. Deficiencies in Kv7 subunits, similar to Kv7.2 subunit mutations, can lead to epilepsy. Kv7 channels reduce neuronal hyperexcitability by activating voltage-gated potassium channels. They also inhibit repeated depolarization of action potentials. Mutations in genes encoding potassium channels, such as *KCNQ2* and *KCNQ3*, can result in reduced potassium ion flow. This leads to prolonged depolarization and increased neuronal hyperexcitability, as potassium ion influx occurs only after repolarization of the neuronal membrane following depolarization. These insights highlight the critical role of potassium ion channels in maintaining proper neuronal excitability and the consequences of their dysfunction in epilepsy [4, 5, 8, 12, 45].

The role of calcium ions as second messengers is essential for both healthy brain function and the development of various neurological disorders, including epilepsy. Calcium ions enter cells through voltage-gated calcium channels, which are instrumental in transmitting electrical signals and initiating transduction cascades in diverse cell types. Voltage-gated calcium channels are categorized into two main subtypes: low-voltage-activated (LVA) and high-voltage-activated (HVA) channels. These channels allow calcium ions to flow into the intracellular space, where they are utilized for signal transduction. The composition of these channels involves multiple subunits, including α1, β, α2, δ, and γ subunits, within the HVA subtype. The subunits within the HVA subtype, including L-, N-, and P/Q-types, have activation thresholds that generally occur around positive membrane potentials, often up to -20 mV. On the other hand, the LVA channel, or type T, is activated at more positive membrane potentials, reaching up to -70 mV. This particular channel subtype exclusively contains a1 subunits. The transmembrane topology of voltage-gated calcium channels consists of four homologous transmembrane domains, each containing a transmembrane segment and a porous region located between segments S5 and S6. This structural arrangement enables the channel to facilitate the movement of calcium ions across the cell membrane, thereby contributing to various cellular processes and responses. In the context of epilepsy, the dysregulation of calcium ion influx through these channels can disrupt normal brain activity and contribute to the development of seizures and other epileptic phenomena. Understanding the intricate role of voltage-gated calcium channels in neuronal function and their involvement in epilepsy is essential for advancing our knowledge of this complex neurological disorder [45].

Impaired expression of the chloride transporter-like K-Cl cotransporter isoform 2 (KCC2)/NKCC1 complex plays a significant role in epileptogenesis. This transporter system is crucial for regulating the impact of GABA (gamma-aminobutyric acid) on neuronal excitability. GABA, traditionally an inhibitory neurotransmitter, can exert excitatory effects through the K-Cl cotransporter. In mature neurons, GABA typically induces membrane hyperpolarization due to lowered intracellular chloride ion levels. However, during neuronal development, elevated intracellular chloride concentrations can lead to GABA-driven chloride ion efflux and subsequent membrane depolarization. The equilibrium between these opposing effects is tightly controlled by the activity of the K-Cl cotransporter and NKCC1. The balance between KCC2 and NKCC1 activity in neurons dictates whether GABA has a depolarizing or hyperpolarizing effect. In cases of epilepsy, the imbalance in the expression and function of KCC2 and NKCC1 can cause a shift from thetraditional inhibitory effect of GABA to an excitatory one, contributing to hyperexcitability and seizure activity. Notably, in the hippocampus of rat models with epilepsy, there is evidence of increased NKCC1 expression coupled with decreased KCC2 expression. This imbalance likely contributes to the altered neuronal excitability observed in epilepsy. Understanding the intricate interplay between chloride transporters, GABA, and neuronal excitability is essential for unraveling the mechanisms underlying epileptogenesis and potentially identifying therapeutic targets to mitigate the development and progression of epilepsy [12].

## The role of neurogenesis in epileptic seizures and epilepsy

Epileptogenesis is a complex process characterized by the formation of new neurons and circuits within the hippocampal region of the brain. After an acute epileptic seizure, individuals with brain injuries can undergo various types of changes. These changes encompass cellular, structural, and neurochemical alterations [4]. Granular cells located in the dentate gyrus of the hippocampus exhibit continuous proliferation in nearly all mammalian species. However, epilepsy can disrupt this process of new neuronal growth. Interestingly, neurogenesis tends to increase in the aftermath of epileptogenesis. An epilepsy-related disorder in hippocampal neurogenesis encompasses several factors, such as the formation of granular cells in abnormal locations, heightened proliferation of neural cell progenitors, aberrations in morphological features of the neurons, and neuronal hypertrophy [12].

After a sudden seizure, the hippocampus can undergo a range of structural changes, including the degeneration of CA1-CA3 pyramidal neurons and dentate gyrus hilar neurons, as well as the sprouting and abnormal formation of synapses by mossy fibers. Additionally, the loss of inhibitory GABAergic interneurons occurs. During the process of mossy fiber sprouting, axons originating from granular cells in the dentate gyrus establish synapses in unusual locations, particularly in the inner third molecular layer of the gyrus dentatus or the supragranular region. This phenomenon contributes to the synaptic reorganization of mossy fibers. As mossy cells, significant excitatory neurons in the hilus dentatus that usually project to the supragranular area, undergo neuronal death, this structural rearrangement aids in eliminating synapses. Furthermore, the growth of new neural circuits can give rise to recurrent excitatory circuits in dentate granule cells due to axon sprouting and synaptogenesis in the mossy fiber pathway. This increased excitation can contribute to the development of epileptogenesis. Another theory regarding structural reorganization involves functional disconnections between excitatory neurons and dormant (inhibitory) basket cells. According to this theory, GABAergic interneurons, known as basket cells, survive damage induced by epileptogenesis. However, because most excitatory neurons (mossy cells) perish as a result of epileptic seizures, the inhibitory influence of these basket cells diminishes. As a result, these basket cells become inactive and are unable to provide inhibitory feedback to granular cells. This loss of inhibition resulting from the inactivity of basket cells might contribute to the process of epileptogenesis [4].

Acute epileptic seizures, leading to structural alterations, can also enhance the expression of neurotrophic factors and various proteins within the hippocampus. These include sonic hedgehog, brain-derived neurotrophic factor (BDNF), fibroblast growth factor-2 (FGF-2), and nerve growth factor (NGF). The neurotrophin family of proteins, which encompasses FGF-2, BDNF, and NGF, plays a vital role in influencing neuronal survival, differentiation, development, synaptic plasticity, and excitability. Additionally, vascular endothelial growth factors (VEGF) are responsible for promoting angiogenesis. However, elevated levels of VEGF can disrupt the BBB, potentially leading to brain inflammation. Notably, abnormal cerebral angiogenesis can manifest as increased permeability to immunoglobulin G and the loss of tight junctions. Furthermore, the sonic hedgehog protein, which is secreted, regulates the growth and survival of both neuronal and glial progenitor cells. An elevation in the expression of this protein may contribute to the generation of neurons within the hippocampus [4, 17].

## The role of oxidative stress in epileptic seizure

Oxidative stress plays a critical role in the initiation and progression of epilepsy, stemming from the excessive release of free radicals. Experimental animal models, such as the administration of kainic acid and pilocarpine, induce this condition. In response, antioxidant therapy has gained acceptance as a strategy to mitigate oxidative stress and potentially serve as a treatment for epilepsy. However, it’s important to note that not all types of seizures exhibit the same degree of oxidative damage. The impact of oxidative stress varies among seizure types. Compromised antioxidant systems, mitochondrial dysfunction, redox-active metals, activation of the arachidonic acid pathway, and even aging can contribute to oxidative stress, thereby becoming factors in the development of epileptic seizures. Superoxide radicals are implicated in both excitotoxicity and the mediation of oxidative stress. This understanding has prompted the exploration of antioxidant approaches targeting oxidative stress as potential avenues for treating epileptic seizures. Such methods not only hold promise for managing seizures but also offer potential as neuroprotective strategies in epilepsy cases. By addressing the underlying oxidative stress, these interventions may contribute to improving the management and outcomes of epilepsy [8, 12].

The activation of intracellular oxidant systems that rely on calcium ions, such as NADPH oxidase 2 (NOX2), plays a pivotal role in promoting superoxide generation. Notably, NOX2 is regulated by NMDA receptors, highlighting its significance in the context of neuronal hyperexcitability. This phenomenon, observed in the early stages of epilepsy, can lead to chronic epilepsy by inducing enduring neural damage. The interplay between these factors contributes to the complex pathophysiology of epilepsy. Neural networks that govern intricate processes in self-regulatory homeostasis, including the bioenergetic system, become disrupted. This imbalance between excitatory and inhibitory signals, combined with oxidative stress, gives rise to abnormal patterns of activity within neural circuits. These complex interactions contribute to the intricate nature of the progression of epilepsy, as well as its long-term effects on neural function and overall brain health [6].

## The role of gene and protein regulation in epileptic seizure and epilepsy

Brain injury triggers alterations in gene expression, a process largely governed by transcription factors. Within the context of epileptic seizures, the pathophysiology is believed to involve immediate early genes (IEGs) or inducible transcription factors that play a pivotal role in orchestrating cellular responses. Notably, members of the Jun family (such as c-jun, junB, and junD) and the Fos family (including c-fos, fosB, fra-1, and fra-2) are implicated in this mechanism. Both gene families encode crucial transcription factors, including c-fos and c-jun, which are integral components of the transcription factor activator protein-1. During epileptic seizures, hippocampal neurons have been observed to display heightened mRNA expression of these factors. The precise functions of IEGs in mediating early genes during epileptogenesis remain to be fully elucidated, generating ongoing uncertainty. Broadly, transcription factors from the Fos and Jun families play roles in gene transcription, cell proliferation, regeneration, and cell death within the central nervous system. The expression of IEGs represents a critical step in the biological cascade leading to neuronal apoptosis, ultimately resulting in cell death. Understanding the intricate interplay between these transcription factors and their influence on cellular processes contributes to unraveling the complex molecular landscape underlying epileptic seizures and their aftermath [4].

Inducible cyclic adenosine monophosphate (cAMP) early repressor (ICER), another transcription factor, emerges as a significant player in the intricacies of epileptogenesis. A class of proteins collectively known as ICER arises from the cAMP-responsive element modulator (CREM) gene. Additionally, ICER mRNAs are generated by the internal promoter of CREM. The central role of ICER lies in its ability to inhibit the cAMP-responsive element (CRE)-mediated transcription of genes. ICER exerts its influence by suppressing the transcription of genes that rely on CRE-mediated mechanisms. A key aspect of its function is the inhibition of the activity of the transcription factor cAMP-responsive element binding protein (CREB). This unique regulatory action positions ICER as a pivotal player in governing both neuronal plasticity and programmed cell death within the nervous system. Notably, CREB is responsible for activating CRE transcription, which holds paramount importance for maintaining neuronal survival. The impact of ICER on neuronal cultures reveals an apoptotic effect through its overexpression in these contexts. Furthermore, the heightened expression of ICER in neurons following excitatory inputs suggests its involvement in apoptotic processes. Yet, despite ongoing research efforts and some divergent findings, the precise role of ICER in the complex web of epileptogenesis remains elusive. As the scientific community continues to explore this intricate topic, elucidating the specific contributions of ICER to the development of epilepsy holds the promise of enhancing our understanding of the underlying mechanisms and potentially paving the way for innovative therapeutic strategies [4, 9, 46].

A multitude of studies have delved into the impact of epileptic seizures on the expression of neuropeptides within the hippocampus. Among the neuropeptides that have garnered attention in these studies are cortistatin, galanin, neuropeptide Y (NPY), thyrotropin-releasing hormone, cholecystokinin, and neurokinin B [47–49]. NPY is localized on GABAergic interneurons within the hippocampus and serves as an inhibitor of excitable neurons. Experimental studies involving models of epileptic seizures have highlighted intriguing findings regarding NPY’s expression. Overexpression of NPY and its corresponding mRNA within the hippocampus, as well as aberrant NPY expression in hippocampal granule cells and mossy fibers—cellular regions not typically associated with this neuropeptide—have been reported in these investigations [50, 51]. The outcomes of this study underscore an inherent adaptive mechanism that counteracts the hyperexcitable state induced by epileptic episode stimulation. Further support for this notion is provided by investigations showcasing diminished occurrences of spontaneous epileptic seizures and the prevention of epileptogenesis through NPY gene therapy in experimental animal models. Galanin, on the other hand, functions as a modulator of epileptic seizures by activating galanin receptors GalR1 and GalR2. This action enables galanin to sustain a delicate equilibrium between glutamatergic excitation and galaninergic inhibition within the hippocampal gyrus dentatus [4].

## The role of thalamocortical circuit disruption in epilepsy, especially the absence type

The disruption of the circuitry connecting the thalamus and the cerebral cortex constitutes the driving mechanism behind absence epilepsy. This circuit plays a pivotal role in orchestrating the rhythmic excitation of the cortex by the thalamus during normal sleep cycles. Within this circuit, three distinct populations of neurons are involved: thalamic relay neurons, thalamic reticular neurons, and cortical pyramidal neurons. Thalamic relay neurons possess the capability to activate cortical pyramidal neurons during wakefulness and rapid eye movement (REM) sleep phases. The underlying electrical activity is likely facilitated by T-type calcium ion channels, which elicit low-threshold depolarization and initiate action potentials through voltage-gated sodium channels. Thalamic reticular neurons, through their inputs, exert control over the activation of thalamocortical circuits by inducing hyperpolarization in relay neurons, thereby generating electrical activity. The mutual inhibition of reticular neurons is achieved through collateral networks stemming from adjacent reticular neurons. Completing this circuit, both cortical pyramidal and thalamic relay neurons project to reticular neurons. Notably, this circuit’s dynamics can be modulated by ascending noradrenergic, serotonergic, and dopaminergic inputs to the thalamus, potentially intensifying the electrical activity. During the non-REM sleep phase, depolarized thalamic relay neurons synchronously and rhythmically stimulate the cortex, resulting in the generation of sleep spindles—distinct brain wave patterns.

In contrast to the activity observed during wakefulness, where thalamic relay neurons are depolarized and sensory information is conveyed to the brain in a non-rhythmic manner, the functioning of thalamocortical circuits during sleep follows a distinct pattern. In the context of absence epilepsy, faulty circuitry generates rhythmic activation of the cortex during waking hours, akin to the pattern observed during typical non-REM sleep phases. This aberrant activity gives rise to the characteristic brain wave patterns and clinical manifestations of absence epilepsy. Several potential factors contribute to these circuitry abnormalities, including disruptions in modulatory input from the brainstem, the role of T-type calcium ion channels, alterations in GABA receptor function, or a combination of these factors. However, the precise nature of the circuitry malfunction remains to be definitively identified. An alternate perspective posits that abnormal paroxysmal episodes of rhythmic cortical activation (absence epileptic seizures) could arise from an underlying neural circuit dysfunction that mimics the physiological state of rhythmic cortical activation seen during sleep [10].

Nowadays, research has showed that the hippocampus circuit plays a crucial role in comprehending the intricacies of epilepsy, particularly the absence type. The hippocampus that works in memory and spatial navigation also has complicated connections with the cortex that contribute to brain activity regulation. The trisynaptic circuit is a critical channel for information processing inside the hippocampus formation. The dentate gyrus, the CA3 area, the CA1 region, and the subiculum are all part of this circuit. At each of these steps, excitatory glutamatergic neurons create synapses, allowing information to be sent sequentially. The circuit’s unique design allows for the modification of incoming signals and the encoding of memories. The synchronization of neuronal activity inside the hippocampus is crucial for maintaining optimal brain function. Disruptions in these coordinated rhythms might result in seizure activity. Incorporating the hippocampus into the knowledge of absence epilepsy provides a more comprehensive view of the neuronal networks involved in this condition. The interconnection of the hippocampus and cortex enables not only for the transmission of spatial and contextual information, but also for the control of general brain states, including those linked with seizures. Considering hippocampus circuitry in addition to the thalamocortical circuit gives a more thorough framework for appreciating the complicated web of connections involved in absence epilepsy. The hippocampus may play a role in the rhythmic cortical activity seen during absence seizures, working in tandem with the damaged thalamocortical circuit to generate an environment favourable to aberrant synchronized firing.

## The role of miRNAs as biomarkers in epilepsy and epileptogenesis

Epigenetic mechanisms that are not linked to changes in DNA sequence but rather to transcription or post-transcriptional regulation can play a role in epileptogenesis. These mechanisms influence various essential biological pathways, some of which are modulated by miRNAs, and are associated with the development of various types of epilepsy [37, 52]. MicroRNAs (miRNAs) serve as potential regulatory mechanisms and therapeutic targets for epilepsy by modulating mRNA stability and translational processes, thereby inhibiting the expression of numerous proteins. These miRNAs hold promise as both biomarkers and targets for therapeutic interventions, as they reflect the intricate gene expression regulation associated with epilepsy. Altered expression of specific miRNAs has been observed, such as serum miRNA-4521 in the hippocampus of individuals with temporal lobe epilepsy, as well as changes in brain tissue in experimental animal models of SE [53–56]. Following epileptic episodes, alterations were observed in the expression levels of certain miRNAs, including those associated with transcription factors and elements involved in neurotransmitter signaling. Changes in miRNA levels in the bloodstream suggest their potential utility as diagnostic biomarkers. Earlier studies primarily focused on the regulatory impact of miRNAs on their target genes. Some investigations explored the consequences of altered gene expression on individual miRNAs, observing fluctuations in miRNA levels in experimental mouse or rat models, and tracking the progression of epilepsy-related pathology. Recent advances in epigenetic profiling are shedding light on the potential role of miRNAs in the pathophysiology of epilepsy. Methylation status and miRNA expression correlations have been identified in hippocampal samples from both human subjects and mouse models. MiRNAs are abundantly present in the brain and play a crucial role in regulating neurogenesis [37, 57, 58].

Numerous types of miRNAs are expressed in response to epileptic conditions. Among them, miR-146a is notable, as it has been observed to exhibit elevated expression in both humans with temporal lobe epilepsy and mice with epilepsy. Sense and antisense oligonucleotides, referred to as agomirs (mimics) and antagomirs, respectively, can efficiently modify the activity of miRNAs. This manipulation of miRNA function can lead to changes in gene expression, offering a promising avenue for the exploration of novel treatment targets for epilepsy [59]. Upon initial investigation in individuals with epilepsy, miR-146a, a miRNA known to regulate inflammatory responses, exhibited an increased presence in the hippocampus [37]. MicroRNA-134 (miR-134), a widely expressed miRNA in brain neurons, plays a role in controlling the formation of dendritic spines by inhibiting LIM domain kinase 1 (LIMK1) and other targets. This function is particularly relevant as dendritic spine reorganization is a feature observed in experimental human epilepsy and is crucial for intercellular excitatory communication, especially in hyperexcitability disorders like epilepsy. In a study conducted by Reschke et al. [60], miR-134 was identified as one of the miRNAs exhibiting increased expression in hippocampal regions affected during SE induced by intra-amygdala kainic acid administration in mice. Furthermore, miR-134 was found to be overexpressed in human neocortex resection samples from patients with refractory temporal lobe epilepsy. The upregulation of miR-134 expression was also observed in both in vitro and in vivo models of neuronal stimulation and epileptic seizures. Remarkably, intracerebroventricular administration of cholesterol-tagged locked nucleic acid (LNA) antagomirs (Ant-134) effectively reduced miR-134 levels for an extended period of at least one month in mice experiencing epileptic seizures resistant to the convulsant effects of kainic acid and pilocarpine hydrochloride. This intervention highlights the potential of targeting miR-134 for therapeutic purposes in epilepsy [60].

MiRNAs such as miR-146a, miR-134, miR-132, miR-128, miR-34a, miR-324-5p, miR-124, miR-155, miR-142-5p, miR-21-5p, miR-187, and miR-106b-5p have all been implicated in the process of epileptogenesis. Among these, miR-106b-5p stands out as a particularly accurate predictor of epileptogenesis, as suggested by Tiwari et al. [59]. Studies conducted by Cava et al. [37] have also shown that miR-106b-5p is upregulated in epileptic conditions. The expression of circulating miRNAs shows dynamic variations in connection with the pathological process of epilepsy. Some miRNAs can be rapidly released following an epileptic seizure, while others may not be expressed until the condition progresses to a chronic stage. Over the past five years, research by Wang et al. [55] has led to the discovery of around 100 distinct miRNAs in both epileptic patients and animal models. This extensive research provides compelling evidence for the significant changes in miRNA expression associated with epilepsy. Although several miRNAs have been linked to epilepsy, further investigations are necessary to determine whether these dysregulated miRNAs can serve as predictive markers for epilepsy risk, diagnostic tools, or indicators of disease progression. Wang et al. [55] conducted a study involving 117 epilepsy patients and 112 controls, utilizing serum samples from epilepsy patients. The study revealed that Let-7d-5p, miR-106b-5p, miR-130a-3p, and miR-146a-5p exhibited increased expression compared to controls, while miR-15a-5p and miR-194-5p showed decreased expression (*p* < 0.0001). Notably, miR-106b-5p in serum demonstrated the highest diagnostic value for epilepsy, with a sensitivity of 80.3% and specificity of 81.2% [55].

In contrast to therapy-responsive groups, a study conducted by Alsharafi et al. [61] highlighted that another investigation identified altered miRNA expression patterns in drug-resistant individuals. Among the findings, 12 miRNAs exhibited decreased expression, while three miRNAs showed increased expression in this drug-resistant group. In comparison to both the treatment-responsive group and the control group, certain miRNAs confirmed through quantitative real-time polymerase chain reaction (qRT-PCR) analysis, including miR-194-5p, miR-301a-3p, miR-30b-5p, miR-342-5p, and miR-4446-3p, displayed statistically significant dysregulation in the drug-resistant group. In this context, miR-301a-3p emerged as a standout diagnostic biomarker for drug-resistant epilepsy among these five miRNAs, demonstrating high levels of both sensitivity and specificity. Notably, it was observed that patients with temporal lobe epilepsy exhibited lower expression levels of the miRNAs miR-301a-3p and miR-106b-5p within their hippocampal tissue [61].

In a study conducted by An et al. in 2016, the roles of eight circulating miRNAs associated with epilepsy were investigated in relation to various cellular processes linked to epilepsy [62]. Through in silico analysis, the study revealed distinct pathways influenced by these miRNAs. Firstly, the “immune system” pathway was highlighted, particularly in the context of neuro-inflammation involving microglia. MiRNAs including miR-15a-5p (with decreased expression), miR-106b-5p, miR-146, and miR-451 were found to impact this pathway, resulting in the upregulation of several genes involved in immune response. MiR-106b, which is also associated with inflammation in Alzheimer’s disease, exhibited increased expression in epilepsy. Secondly, the “cell cycle” pathway was identified through in silico analysis, encompassing processes related to neurogenesis, cell cycle regulation, and cell proliferation. This pathway indicated that these miRNAs, specifically miR-15a-5p, miR-34a, miR-106b-5p, and miR-146, likely influence genes involved in neuronal cell differentiation and proliferation. Notably, miR-106b-5p displayed heightened expression in the early stages of an epilepsy mouse model induced by electrical stimulation. This suggests that this miRNA could potentially contribute to the interruption of neuronal cell cycle progression and subsequent neuronal death. Thirdly, the study identified confirmed targets of miR-15a-5p (with reduced expression), miR-106b-5p, miR-146, and miR-45 that are implicated in both pro- and anti-apoptotic signaling pathways. This suggests a complex role for these miRNAs in regulating apoptosis, which is a significant process in the pathophysiology of epilepsy. Overall, this study sheds light on the intricate involvement of specific miRNAs in various cellular processes related to epilepsy, further enhancing our understanding of the molecular mechanisms underlying this disorder [37].

## Conclusions

The advancement of molecular science has provided valuable insights into the understanding of epilepsy. The pathophysiology of epileptogenesis, the process leading to epilepsy, is intricate and involves the interplay of various factors such as neurotransmitters, ion channels, receptors, neurogenesis, oxidative stress, inflammation, apoptosis, hyperactivation of the mTOR signaling pathway, and the regulation of numerous genes and proteins. Epileptic seizures arise from a sudden imbalance between excitatory and inhibitory neurotransmitters. Inflammation and oxidative stress can occur both prior to and following epileptic convulsions. Disruption of calcium ion homeostasis due to excitotoxicity can lead to cell death through apoptosis or necrosis pathways. The process of epileptogenesis, marked by diverse structural, neurochemical, and cellular changes, is also influenced by hippocampal neurogenesis. Further exacerbating brain damage and increasing the risk of recurrent epileptic seizures are glial cell alterations and modifications to the BBB. Recent research has highlighted the role of specific microRNAs (miRNAs) such as miR-146a, miR-134, miR-132, miR-128, miR-34a, miR-324-5p, miR-124, miR-155, miR-142-5p, miR-21-5p, miR-187, and miR-106b-5p in epileptic disorders. However, a deeper investigation is needed to fully understand the individual contributions of each miRNA to epilepsy. Encouragingly, emerging evidence supports the potential of utilizing microRNA-induced silencing as a therapeutic target in epilepsy. Addressing the challenges in pinpointing the target and cell specificity of miRNAs and developing safe and effective strategies to modulate miRNAs will be pivotal in translating these promising findings into clinical applications for the treatment of epilepsy or the prevention of epileptogenesis.

## Data Availability

Availability of data and materials is not applicable in this study.

## Abbreviations

AMPA: α-Amino-3-hydroxy-5-methyl-4-isoxazolepropionic acid
ATP: Adenosine triphosphate
BBB: Blood-brain barrier
cAMP: Cyclic adenosine monophosphate
ESAM: Endothelial selective adhesion molecules
FCD: Focal cortical dysplasia
GABA: γ-Amino butyric acid
HCN: Hyperpolarization-activated cyclic nucleotide-gated
HMGB1: High-mobility group box 1
ICE: Interleukin-1β converting enzyme
ICER: Inducible cAMP early repressor
IL-1: Interleukin-1
IL-6: Interleukin-6
IL-1RI: Interleukin-1 receptor type I
JAMs: Junctional adhesion molecules
LD: Lafora disease
LVA: Low-voltage-activated
mTOR: Mammalian target of rapamycin
NKCC1: Na-K-2Cl cotransporter isoform 1
NMDA: N-methyl-D-aspartate
NMDAR: N-methyl-D-aspartate receptors
PDS: Paroxysmal depolarization shifts
P-gp: P-glycoprotein
PIK3CA: Phosphatidylinositol-4,5-bisphosphate 3-kinase-α
PTEN: Phosphatase and tensin homolog
SE: Status epilepticus
STRADA: STE20-related kinase adaptor-α
TNFR1: Tumor necrosis factor receptor 1
TSC: Tuberous sclerosis complex
