# Correction: Biomolecular mechanisms of epileptic seizures and epilepsy: a review

**DOI:** 10.1186/s42494-024-00157-4

**Published:** 2024-04-12

**Authors:** Komang Trisna Sumadewi, Saktivi Harkitasari, David Christopher Tjandra

**Affiliations:** 1 Anatomy-Histology Department, Faculty of Medicine and Health Science, Warmadewa University, Denpasar, 80235 Indonesia; 2 Neurology Department, Faculty of Medicine and Health Science, Warmadewa University, Denpasar, 80235 Indonesia; 3 Bachelor of Medicine Study Program, Faculty of Medicine, Udayana University, Denpasar, 80234 Indonesia

**Correction** **:** **Acta Epileptol 5, 28 (2023)**

**https://doi.org/10.1186/s42494-023-00137-0**

Following publication of the original article [1], the authors mistakenly duplicated sections "Sodium ion channels" and "Potassium ion channels" in the article.

The duplicated sections have been removed.

The original article [1] has been updated.
